# Left and right corticobasal syndrome: comparison of cognitive profiles between metabolic imaging - matched groups

**DOI:** 10.1007/s10072-023-07148-2

**Published:** 2023-10-27

**Authors:** Valeria Isella, Daniele Licciardo, Francesca Ferri, Cinzia Crivellaro, Sabrina Morzenti, Ildebrando Marco Appollonio, Carlo Ferrarese

**Affiliations:** 1 School of Medicine and Surgery (Neurology), University of Milano-Bicocca, Via Cadore 48, 20900 Monza(MB), Italy; 2 Fondazione IRCCS San Gerardo Dei Tintori (Neurology), Monza, Italy; 3 Fondazione IRCCS San Gerardo Dei Tintori (Nuclear Medicine), Monza, Italy; 4 Fondazione IRCCS San Gerardo Dei Tintori (Medical Physics), Monza, Italy

**Keywords:** Corticobasal syndrome, Movement disorders, FDG-PET, Dementia, Parkinsonism, Cognition

## Abstract

**Background:**

Corticobasal syndrome (CBS) is typically asymmetric. Case reports suggest that left-hemisphere CBS (lhCBS) is associated with major language impairment, and right-hemisphere CBS (rhCBS) is associated with major visuospatial deficits, but no *group* study has ever verified these observations. In our study, we enrolled 49 patients with CBS, classified them as lhCBS or rhCBS based on asymmetry of hypometabolism on brain FDG-PET and compared their cognitive and behavioural profiles.

**Methods:**

We defined asymmetry of hypometabolism upon visual inspection of qualitative PET images and confirmed it through paired comparison of left- and right-hemisphere FDG uptake values. The two groups were also matched for severity of hypometabolism within the more affected and more preserved hemispheres, to unravel differences in the cognitive profiles ascribable specifically to each hemisphere’s functional specializations. All patients were assessed for memory, language, executive and visuospatial deficits, apraxia, neglect, dyscalculia, agraphia and behavioural disturbances.

**Results:**

LhCBS (n. 26) and rhCBS (n. 23) patients did not differ for demographics, disease duration and severity of global cognitive impairment. The two cognitive profiles were largely overlapping, with two exceptions: Digit span forward was poorer in lhCBS, and visual neglect was more frequent in rhCBS.

**Conclusions:**

After balancing out patients for hemispheric hypometabolism, we did not confirm worse language or visuospatial deficits in, respectively, lhCBS and rhCBS. However, verbal short-term memory was more impaired in lhCBS, and spatial attention was more impaired in rhCBS. Both of these functions reflect the functional specialization of the left and right fronto-parietal pathways, i.e. of the main loci of neurodegeneration in CBS.

## Introduction

Corticobasal syndrome (CBS) is a rare neurodegenerative motor and cognitive disorder that has several possible underlying pathologies, e.g. corticobasal *degeneration* (a 4R tauopathy) and Alzheimer’s disease, and involves the brainstem, basal ganglia and neocortex, especially at the level of the fronto-parietal regions. Its clinical presentation is characterized by various motor, cortical sensory, cognitive and behavioural disturbances including extrapyramidal signs and symptoms, cortical sensory loss, limb apraxia, and linguistic, frontal-executive and visuospatial deficits [1–4].

A very typical feature of CBS is asymmetry, i.e. the predominantly left or predominantly right distribution of neurodegenerative processes in cortical and subcortical structures evident at autopsy and on structural and functional neuroimaging and associated with motor and sensory-motor symptoms (akinetic-rigid syndrome, myoclonus, dystonia, limb apraxia, alien hand, cortical sensory loss) more severe in the body side opposite the more affected hemisphere [1–4]. Although this feature is considered specific of CBS to the point of being included in standardized diagnostic criteria [5, 6], its impact on the cognitive manifestations of the disease has never been investigated in a formal group study. Case reports and case series support the view that CBS presenting with worse neurological signs and symptoms at the level of the right extremities is associated with major language impairment [3], whereas CBS affecting predominantly left extremities shows major visuospatial deficits [3, 7], but empirical studies comparing the cognitive profile of sizeable groups of patients with left-hemisphere or right-hemisphere CBS (lhCBS, rhCBS) are lacking. The present work aimed at filling this gap in the literature and at increasing knowledge about the neuropsychological presentation of CBS.

‘Asymmetry’ conventionally refers to the somatic distribution of neurological signs typical of CBS, which mirrors brain asymmetry. However, clinical features are an indirect index of neurodegenerative damage in the neocortex, and features like parkinsonism, alien hand or cortical sensory loss are difficult to rate quantitatively and in a fine-grained and objective manner. Furthermore, in some predominantly cognitive CBS phenotypes, like behavioural/dysexecutive and visuospatial syndromes, motor features may be absent. We therefore chose to define hemispheric asymmetry using metabolic neuroimaging. Participants underwent brain PET with 18-fluoro-deoxy-glucose (FDG), and we then extracted values of FDG uptake for both hemispheres from individual scans with a software for the automated analysis of metabolic images. Using this quantitative and objective measure of brain metabolism allowed us to achieve three goals. First, we confirmed through statistical analysis on uptake values that the two groups were significantly asymmetric. Second, we matched the two groups for the severity of involvement of the least affected hemisphere, i.e. the right hemisphere in lhCBS and the left hemisphere in rhCBS. Since neurodegenerative disorders may be asymmetric, but are virtually always bilateral, the two groups had to be balanced also for damage within the least affected hemisphere. Third, we matched carefully left and right patients for severity of neurodegenerative damage also within the more affected hemisphere. This matching was necessary for disentangling cognitive dissimilarities linked to each hemisphere’s functional specialization, from those linked to disproportion in degenerative burden between the two groups.

The neuropsychological protocol used for outlining the cognitive profile of left and right cases included measures aimed at detecting deficits in the main cognitive and behavioural domains (short-term and long-term memory, language production and comprehension, attention and executive functions, visuo-spatial abilities, praxis, spatial attention, calculation, writing, mood and behaviour). We hypothesized that differences between groups carefully matched for severity of hypometabolism would emerge for deficits associated with dysfunction of the left (e.g. language) or the right (e.g. spatial functions) hemisphere and underpinned by brain regions (e.g. the fronto-parietal cortex) typically involved in CBS.

## Methods

### Participants

Patients were recruited from the memory and movement disorders clinics of Fondazione IRCCS San Gerardo dei Tintori, Monza. Patients’ clinical records were reviewed for neurological signs and symptoms including, in particular, presence and lateralization of an akinetic-rigid syndrome, myoclonus, dystonia, alien hand and cortical sensory loss. Study candidates had to meet standardized criteria for CBS [5], have undergone brain FDG-PET within 6 months of the neuropsychological assessment, and be right-handed. We took into consideration all CBS phenotypes except primary progressive aphasia, which has an obvious association with prominent involvement of the left hemisphere (at least in the vast majority of right-handed individuals).

Exclusion criteria were concomitant neurological disorders (e.g. stroke, brain tumors, brain injury), psychiatric disturbances (including major depression), a history of substance abuse or mental insufficiency and presence of large and/or numerous vascular lesions on brain CT/MRI scan. Rapidly progressive dementia was also an exclusion criterion.

The study was conducted in accordance with the Declaration of Helsinki and approved by our institution’s ethics committee, Comitato Etico Brianza.

### Acquisition of FDG-PET images

Brain FDG-PET scans were performed on a General Electric Discovery LS PET/CT scanner, on average 3.5 ± 2.6 months within the neuropsychological assessment. CT images were acquired to be used for attenuation correction. PET images were acquired over a period of 15 min with a thickness of 3.27 mm and a matrix of 128 × 128 pixels and reconstructed following an ordered subset expectation maximization algorithm.

### Classification and comparison of patients with lhCBS or rhCBS

Regional FDG uptake values were extracted from individual PET scans using Cortex ID Suite, a software for automated analysis of PET images developed and marketed by GE Healthcare (Waukesha, WI, USA) that calculates tracer uptake values normalized by whole brain uptake, for 12 cortical regions of interest (ROIs) in each hemisphere: the lateral and mesial prefrontal cortex, the sensorimotor cortex, the anterior and posterior cingulate and precuneus, the superior and inferior parietal lobe, the lateral and mesial temporal cortex and the lateral occipital and primary visual areas. Uptake values for the 12 left and 12 right ROIs were averaged for obtaining one global index of hemispheric hypometabolism.

Operatively, qualitative PET scans were first assessed visually, by a Nuclear Medicine specialist with over 20 years of experience (C.C.), who inspected the images displayed on a terminal that permitted to manipulate orientation (axial, coronal, sagittal) and colour scale and classified hypometabolism as symmetric / asymmetric left > right / asymmetric right > left. Asymmetry was then verified statistically through paired comparison of mean FDG uptake values for the left and right hemisphere within each of the two CBS groups. Hypometabolism was expected to be significantly worse in the left than the right hemisphere for the lhCBS group and significantly worse in the right than the left hemisphere for the rhCBS group.

As to matching of lhCBS and rhCBS groups for overall severity of hypometabolism, it was achieved by contrasting the two groups for left- and right-hemisphere mean uptake values with independent Student’s *t* test. The following criteria had to be met: left hypometabolism in lhCBS patients was expected to be comparable to right hypometabolism in rhCBS patients; right hypometabolism in lhCBS patients was expected to be comparable to left hypometabolism in rhCBS patients; left hypometabolism in lhCBS patients was expected to be worse than left hypometabolism in rhCBS patients, and vice versa.

### Neuropsychological assessment

The cognitive profile was evaluated with an extensive battery of standardized neuropsychological tests. Specifically, selective attention was assessed with Attentional Matrices, short-term memory with the Digit span forward, long-term memory with the Rey Auditory Verbal Learning Test (RAVLT) and recall of Rey-Osterrieth Complex Figure (ROCF), language production with Category fluency and a Picture naming test, visuo-constructional abilities with copy of ROCF, limb apraxia with De Renzi’s test of Ideomotor Apraxia and executive functions with Letter fluency and the Frontal Assessment Battery (FAB). Verbal comprehension was evaluated with different tests, namely either the Token test or the Sentence Comprehension subtest of the Neuropsychological Examination of Aphasia (ENPA) battery; in order for the two measures to be *comparable*, *raw scores* were converted into zeta-scores. We also assessed the presence/absence of visual neglect, acalculia and agraphia. Neglect was identified on inspection of figure copies (ROCF and MMSE pentagons) considering lateralized omissions, without using a quantitative measure, while acalculia and agraphia were established with MMSE subtests of, respectively, 7s serial subtraction and writing of a sentence.

The neuropsychological battery also included the Mini-Mental State Examination (MMSE) as a measure of global cognition and the Neuropsychiatric Inventory (NPI) for the assessment of mood and behaviour.

### Statistical analysis

Statistical analysis was performed using SPSS 28.0 (SPSS Statistics for Windows, Armonk, NY: IBM Corp.). Data were normally distributed. Paired or independent Student’s *t* tests were used to compare continuous variables within and between the two groups, and Fisher’s exact test was used for comparing categorical variables between the two groups. Threshold for significance was set at *p* < 0.05.

## Results

### General characteristics of study participants

A total of 58 patients fulfilled criteria for participating in the study. Nine (15.5%) showed substantially symmetric hypometabolism on FDG-PET by visual rating and were excluded from the analyses. Twenty-six of the 49 asymmetric cases (53.1%) showed prominent left-hemisphere hypometabolism and 23 (46.9%) prominent right-hemisphere hypometabolism.

**Fig. 1:**
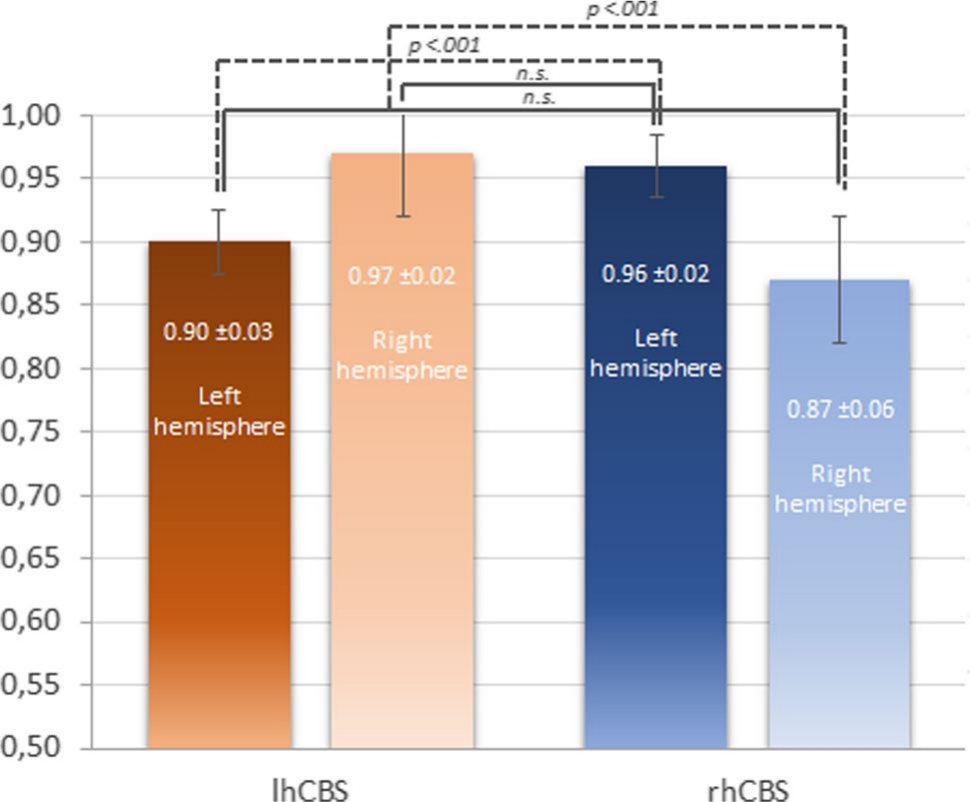
Comparison of mean hemispheric normalized FDG-uptake values between left-hemisphere CBS (lhCBS) and right-hemisphere CSB (rhCBS). Thin lines indicate the paired contrast between the two hemispheres within each of the two groups; thick lines indicate the inter-group contrast between the more affected hemispheres and between the more preserved hemispheres; dotted lines indicate the inter-group contrast between the more affected hemisphere of one group and the more preserved hemisphere in the other group

Statistical comparison of FDG uptake values confirmed the metabolic asymmetry in both groups and demonstrated that the two groups did not differ for severity of hypometabolism in their more affected hemispheres, as well as in their more preserved hemispheres, and showed more severe hypometabolism in their affected hemisphere than in the other group’s preserved hemisphere (Fig. 1).

In 38/49 cases (77.6%), metabolic asymmetry was paralleled by neurological asymmetry, i.e. more severe left-hemisphere hypometabolism was paired with worse motor or sensory-motor symptoms on the right body side, and vice versa. Only in one rhCBS patient (2.0%) neurological manifestations were more severe ipsilaterally to the hemisphere with worse hypometabolism (right body side). In all the other cases, neurological signs were symmetric (n. 6, 12.2%) or absent (n. 4, 8.2%).

Comparison of socio-demographic characteristics, disease duration and global severity of cognitive impairment as measured by MMSE did not show statistically significant differences between the two groups (Table 1).

**Table 1:** Participants’ socio-demographic and clinical features

	Whole sample	lhCBS	rhCBS	*p*
	n. 49	n. 26	n. 23	
Age	71.6 ± 6.3	72.1 ± 5.7	70.9 ± 7.0	0.515
Sex—men	24 (49.0)	13 (50.0)	11 (47.8)	1.000
Education (years)	8.2 ± 3.8	8.0 ± 3.8	8.4 ± 3.8	0.777
Disease duration (years)	2.1 ± 1.0	2.0 ± 1.1	2.1 ± 0.9	0.841
Mini-Mental State Examination	21.0 ± 4.1	21.0 ± 4.2	20.9 ± 4.1	0.917

Data are n. (%) or mean ± standard deviation

### Comparison of cognitive profiles of lhCBS and rhCBS

**Fig. 2:**
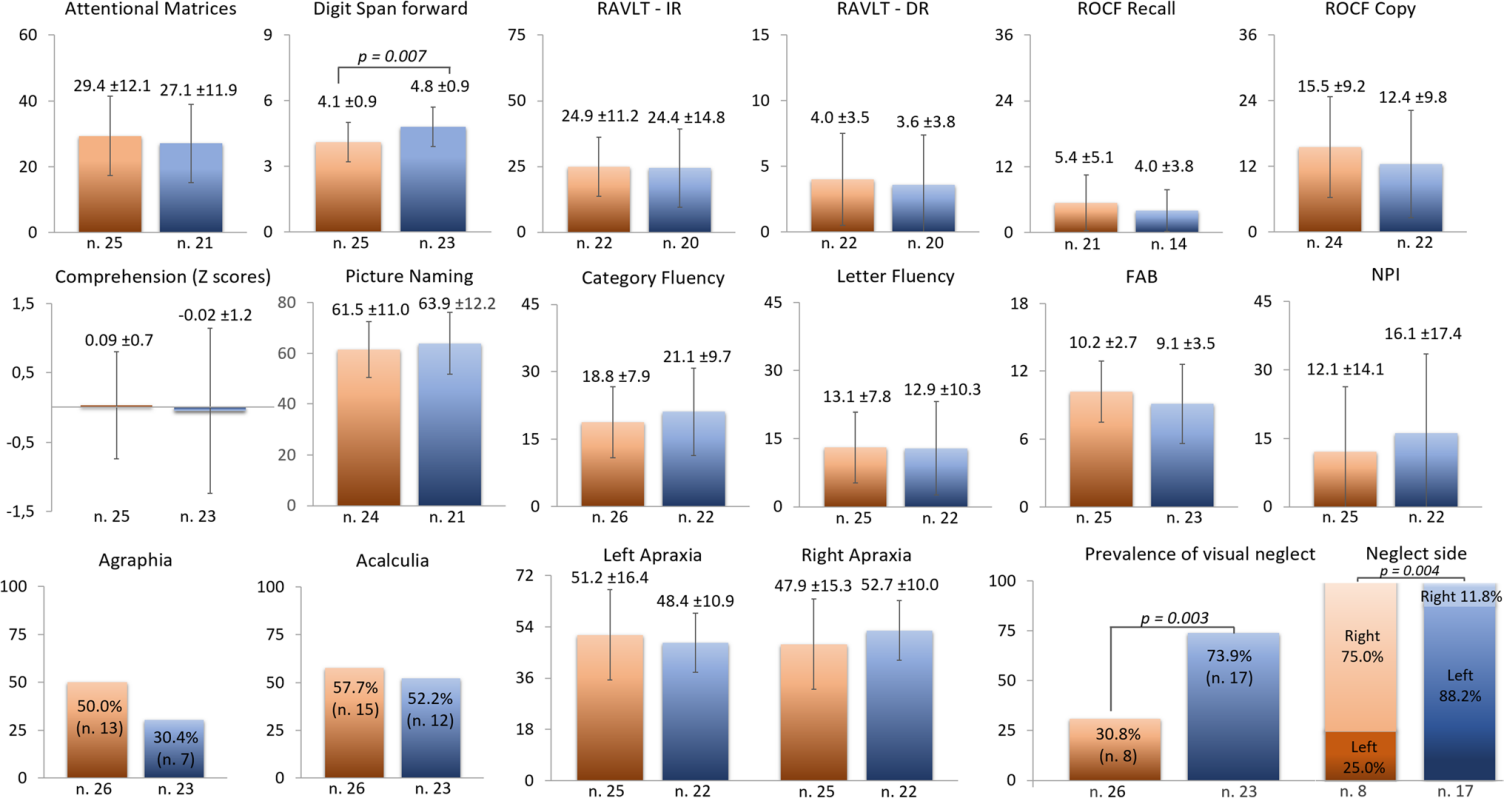
Comparison of neuropsychological measures between left-hemisphere CBS (orange bars) and right-hemisphere CSB (blue bars). For all tests, higher scores indicate better performance except the NPI (higher scores indicate more severe neuropsychiatric disturbances). Comprehension zeta scores were derived from raw scores of the Token test or the Sentence Comprehension subtest of the Neuropsychological Examination of Aphasia (ENPA) battery. FAB, Frontal Assessment Battery; NPI, neuropsychiatric inventory; RAVLT–DR, Rey Auditory Verbal Learning Test Delayed Recall; RAVLT–IR, Rey Auditory Verbal Learning Test Immediate Recall; ROCF, Rey-Osterrieth Complex Figure

We found no significant intergroup difference on measures of long-term memory, language, attention, executive functions, visuoconstructional abilities, limb apraxia, acalculia, agraphia or neuropsychiatric disturbances (Fig. 2). Only two cognitive domains were differentially impaired in the two groups: scores on the verbal short-term memory test Digit span forward were poorer in lhCBS than rhCBS (*p* = 0.007), while visual neglect was more frequent in rhCBS than lhCBS patients (*p* = 0.003) and more frequently left-sided in rhCBS and right-sided in lhCBS (*p* = 0.004) (Fig. 2).

## Discussion

Our study was aimed at investigating the presence of differences in the neuropsychological profile of CBS patients with prominent hypometabolism of the left or right hemisphere. Crucially, in our lhCBS and rhCBS groups, we verified formally that hypometabolism in the more affected hemisphere was significantly worse than in the opposite hemisphere (i.e. patients were truly asymmetric), and severity of damage was well balanced between the two groups at the level of both the more affected and the more preserved hemisphere. This approach permitted to control for the fact that neurodegeneration affects both brain sides in CBS and to unravel cognitive differences ascribable unambiguously to the functional specialization of each hemisphere, rather than to disparities in the severity of degeneration between the two groups.

We anticipated that differences would emerge for cognitive domains whose neuroanatomical underpinnings are functionally dominant in one hemisphere (as for verbal functions and the left hemisphere) and are affected by degeneration in CBS (e.g. the fronto-parietal cortex [1, 2, 4]) but not for domains that are only weakly lateralized to one side of the brain or whose neural substrate is relatively spared in this disorder.

Most of our findings, and in particular those concerning spatial attention and verbal short-term memory, were in agreement with these predictions. We found a higher prevalence of visual neglect in the rhCBS group. We suggest that this was due to the fact that the fronto-parietal regions and connections implicated in the control of spatial attention are functionally asymmetric, with a dominance of the right hemisphere [8] and that these regions are key areas of neurodegeneration in CBS.

Similarly, we hypothesize that results obtained for verbal tasks included in our battery might be accounted for by differences in the degree of lateralization and in the severity of involvement of the dorsal versus ventral streams of language processing. According to the dual stream model of language [9], the dorsal stream that connects the temporo-parietal cortex with the frontal lobe supports auditory-motor integration and phonological buffering for speech production, tapped by verbal short-term memory tasks [10, 11], while the ventral stream running through the temporal lobe subserves concept and lexical retrieval, tapped by naming, fluency and comprehension tasks. While the left and right ventral streams are both actively involved in language processing and highly interacting, the dorsal stream is strongly left-lateralized. The fact that we found a significantly poorer performance of lhCBS patients in a phonological short-term memory test, the Digit span forward, but no intergroup differences for category fluency, picture naming or verbal comprehension, might then reflect a major degree of lateralization and a more severe involvement of the parieto-frontal dorsal stream than of the temporal ventral streams.

The lack of differences in naming or fluency between our two groups seems at odds with the general idea that language is more impaired in left than right CBS. However, this belief was based on informal observation and anecdotal reports and not verified empirically or was referred specifically to the nonfluent aphasia phenotype of CBS [3]. Our analysis showed that, once this phenotype is omitted, the apparent association between more severe language disturbances and lhCBS is not confirmed.

The fronto-parietal cortex is involved also in gesture programming. More precisely, one of the most influential neurocognitive models of action control [12] purports that the dorsal visual stream connecting the superior and inferior parietal lobe to the premotor cortex includes a dorso-dorsal and a ventro-dorsal pathway. The former pathway supports the elaboration of simple, reaching and grasping, movements, while the latter operates skilled tool manipulation. Importantly, the dorso-dorsal pathway is functionally symmetric, while the ventro-dorsal pathway is left-lateralized. In a seminal paper about apraxia in CBS, Stamenova et al. [13] suggested that neurodegeneration along the dorso-dorsal pathway might be the primary source of apraxia in CBS and that, since this stream has a symmetric representation, apraxia ‘would equally affect left and right hemisphere patients’ [13]. Our results are in line with this thesis, as our lhCBS and rhCBS groups were equally impaired on the Ideomotor Apraxia test. This finding is in conflict with evidence from studies on apraxia in the context of stroke, which have consistently shown that this deficit is more likely after a left-sided than a right-sided lesion [14], but is not completely novel in the literature on neurodegenerative disorders, and CBS in particular. Even if some studies have shown an association between apraxia and right-sided motor symptoms [15] and left-hemisphere atrophy [16] in CBS, others have found a similar prevalence of apraxia in patients with left > right atrophy compared with patients with symmetrical atrophy [17] or with right > left atrophy [7], or have shown an association between apraxia scores and bilateral atrophy, at the level of the mesial fronto-parietal or inferior parietal cortex [17, 18]. Future investigations aiming to clarify these discrepant findings should take into account all the types of apraxia that may develop in the course of CBS (not only ideomotor apraxia, but also limb-kinetic and ideational apraxia), which have different neurocognitive substrates and might therefore be differentially affected by asymmetry.

Scores on the FAB, Letter fluency, Attentional Matrices, copy of ROCF and the NPI and prevalence of acalculia were overlapping in our lhCBS and rhCBS patients. We hypothesize that, unlike verbal short-term memory or spatial attention, the neuropsychological domains assessed by these tests are only weakly lateralized to one hemisphere. Executive abilities, attention and neuropsychiatric symptoms are in fact multifaceted neuropsychological constructs with highly distributed anatomical correlates [19–23]. In addition, both the FAB and the NPI are composite scales that provide a single score for multiple processes with different neurocognitive substrates. Perhaps, the use of measures tapping specific, individual executive functions or neuropsychiatric (e.g. frontal) disturbances would have highlighted differences between our two groups. For instance, previous studies in healthy individuals and neurodegenerative patients have shown a relationship between the left frontal lobe and reasoning, fluency and sorting abilities [19, 24] and a relationship between a right fronto-subcortical network and attention [25], motivation [26, 27] and cognitive and emotional inhibition [21, 26, 27]. Likewise, studies on visuo-constructional abilities in clinical and healthy samples have found a link between figure copy tasks and several areas within both hemispheres [28–31], reflecting the multicomponent cognitive nature of copying, and have shown differences in drawing strategy between right- and left-damaged groups (gestaltic in the former and piecemeal in the latter) but not in overall accuracy [29, 32]. Only in one published case series patients with right CBS showed more severe spatial deficits than patients with left CBS, but the right group showed more widespread atrophy on MRI, indicating more severe neurodegeneration [7].

As to calculation, even if it is classically associated with the left inferior parietal cortex [33, 34], this ability is known to recruit also other parietal and frontal areas within both the left and right hemisphere [35, 36]. Furthermore, in our study, we used the serial subtraction item from MMSE for assessing calculation, and subtraction operations have been particularly frequent in patients with right hemisphere damage [35, 36].

The lack of significant differences between our lhCBS and rhCBS groups in the prevalence of agraphia is more difficult to explain in terms of integrity of areas and circuits underlying writing or of an equal contribution of the left and right hemisphere to this ability. In fact, even if the right hemisphere seems to exert some influence on the visuospatial aspects of writing, the key regions for the control of its linguistic and motor aspects belong to the left hemisphere and encompass the fronto-parietal regions consistently involved in CBS (specifically the middle frontal gyrus and angular gyrus[37]). Our tentative explanation is that a more accurate measure of writing ability, more accurate than the MMSE item that we used, would actually confirm the expected major frequency of agraphia in lhCBS.

Our study has some limitations. First of all, the neuropsychological battery was extensive but did not cover all cognitive and behavioural domains, e.g. we had no formal measure of visual neglect, agraphia and acalculia, nor a purely perceptual measure of visuospatial abilities. We cannot rule out that a more thorough investigation of these functions might have revealed additional differences in the clinical presentation of left and right CBS. The battery could also vary slightly across participants (as for verbal comprehension) or be incomplete. The visuospatial memory score, in particular, was unavailable in nearly 40% of rhCBS patients, in most cases because the copy was extremely poor and the recall was not administered. Some neurological data were also missing, as may be the case in retrospective reviews (yet information about motor, sensory-motor and cognitive signs and symptoms was sufficient for applying diagnostic criteria for CBS to all participants). Second, the sample size was relatively small, due to the rarity of CBS, and lacked pathology or biomarker confirmation of the clinical diagnosis. Third, our two CBS groups were matched for severity of hemispheric hypometabolism based on the mean of FDG uptake values of the 12 Cortex ID ROIs, but a more fine-grained matching, e.g. at the lobar level, would have allowed better control of possible focal imbalances. Fourth, we did not take into account the involvement of the basal ganglia. Evidence from studies in healthy and brain damaged subjects has made clear that, in virtue of their connections with the cortex and especially with the limbic system and the frontal lobes, the basal ganglia play a relevant role in the regulation of behaviour and of cognitive functions such as working memory, language, attention, planning and reasoning [38–40]. The accuracy of FDG-PET for subcortical structures is poor, though, and Cortex ID Suite includes only cortical ROIs; thus, we could not explore the contribution of the basal ganglia to the cognitive specificities of left and right CBS. Fifth, average symptoms duration was only 2 years in our sample. While including patients in an early disease stage reduced the risk of clinical overlap due to progressive spreading of neurodegeneration, we cannot rule out that more differences between lhCBS and rhCBS might actually emerge later in the disease course. Finally, since we performed several inter-group comparisons but did not apply corrections for multiple contrasts to threshold for significance, our results should be considered of an exploratory nature.

This is to our knowledge the first group study that compared the cognitive profile of left and right CBS, and that used metabolic imaging for defining and verifying formally the presence of asymmetry, and for matching left and right patients for severity of degenerative damage within the more affected and more preserved hemispheres. We did not confirm the major impairment of language in lhCBS and of visuospatial abilities in rhCBS reported in single cases and case series [3, 7]. Only verbal short-term memory and spatial attention, that is two cognitive functions that are strongly lateralized to one hemisphere and whose neural substrate is affected consistently by neurodegeneration in CBS, were significantly more impaired in patients with, respectively, prominently left and prominently right hypometabolism.

## Data Availability

The data that support the findings of this study are available on request from the corresponding author.
